# Electron Beam Welding Process for Ti6Al-4V Titanium Alloy

**DOI:** 10.3390/ma16145174

**Published:** 2023-07-23

**Authors:** Zbigniew Wencel, Sylwia Wiewiórowska, Paweł Wieczorek, Andrzej Gontarz

**Affiliations:** 1 Pratt & Whitney Kalisz, ul. Elektryczna 4a, 62-800 Kalisz, Poland; zbigniew.wencel@prattwhitney.com; 2 Faculty of Production Engineering and Materials Technology, Czestochowa University of Technology, 42-201 Czestochowa, Poland; pawel.wieczorek@pcz.pl; 3 Faculty of Mechanical Engineering, Lublin University of Technology, 20-618 Lublin, Poland; a.gontarz@pollub.pl

**Keywords:** beam welding, titanium alloy, microhardness, mechanical properties

## Abstract

The electron beam welding process of titanium alloys induces a series of physicochemical changes in the material that remain a relevant and necessary area of investigation. A necessary step performed after the electron beam welding process of titanium alloys in the Ti6Al-4V grade to mitigate the resulting thermal stresses is the post-weld heat-treatment process conducted through stress relieving. This study presents the comparative analysis results of the mechanical properties and structure of the Ti6Al-4V titanium alloy after electron beam welding and subsequent stress-relieving heat treatment at a temperature of 590 °C for 2 h. The analysis focused on the levels of mechanical properties such as microhardness in the heat-affected zone and weld, tensile strength, and microstructure analysis in the heat-affected zone and weld. The aim of the research was to answer the questions regarding whether the post-weld heat treatment through stress relieving after electron beam welding of the Ti6Al-4V titanium alloy would significantly affect the changes in mechanical properties and microstructure of the alloy and whether the applied welding speed in the study would cause a significant depletion of alloying elements in the material. During the course of the study, it was found that conducting the electron beam welding process at a speed of 8 mm/s resulted in a depletion of one of the alloying elements (aluminum) in the face area. However, the decrease in aluminum content was not significant and did not exceed the critical value of 6% specified in the material standards, which determined the material’s application based on its strength properties.

## 1. Introduction

The electron beam welding (EBW) process is characterized by several features: non-contact operation, high process energy, high accuracy, high purity, and the process temperature. This non-contact process enhances precision and prevents distortion of the welded material [1,2,3,4,5].

An electron beam directed at a welded surface possesses extremely high kinetic energy, resulting in the melting and fusion of the metal. The high energy also ensures a rapid welding speed.

EBW is a highly precise process that enables the production of welds with exceptional quality and accuracy. Consequently, it finds extensive applications in industries such as aerospace, space, and medicine [6,7,8,9,10].

This process is conducted under high vacuum conditions, ensuring a clean environment. It demands stringent surface cleanliness, particularly for titanium alloys. The electron beam welding process generates intense heat, causing the metal to evaporate at the point of beam impact, creating the well-known capillary tunnel. During the movement of the beam, the process of joining metals involves closing the capillary tunnel [11,12,13].

Titanium alloys exhibit excellent mechanical properties and corrosion resistance, making them a desirable construction material across various industries. The electron beam welding (EBW) process represents one of the methods employed for welding titanium alloys [14,15,16,17,18,19,20].

The electron beam welding of titanium alloys leads to oversaturation of the welded material’s structure within the weld area, directly affecting the mechanical properties and strength of the joint. Additionally, rapid recrystallization processes that occur during welding introduce significant thermal stresses into the material. Moreover, titanium alloys possess a low coefficient of thermal conductivity (6.70 W/(m·K)), further contributing to the increase in thermal stresses [21,22].

The welding process of titanium alloys results in both structural and geometrical changes, which lead to contraction of the welded component in the weld area. As a consequence, the material experiences structural, thermal, and geometrical stresses. The level of these stresses should be considered comprehensively without dividing them into micro or macro areas. It is known that tensile stresses occur within the weld itself after the welding process due to the presence of the martensitic phase. The direction of these stresses changes as the proportion of the martensitic phase decreases in the alloy structure.

Compressive stresses appear at the boundary of the heat-affected zone. Due to the operational properties of components working under fatigue conditions, it is necessary to reduce the non-uniform stress field in the welded joint through stress relief annealing [23].

The primary challenge in EBW processes for titanium alloys is to prevent excessive porosity in the welded joint, which can occur due to the high welding speed used for thick materials and the titanium’s tendency to adsorb and retain oxide and process impurities on its surface [24].

In this study, the electron beam welding process for Ti6Al-4V grade titanium alloys was examined with a welding speed of 8 mm/s, which is lower than the typical speed [25].

Metallographic studies conducted in this work utilized optical microscopy to determine the shape and size of the weld and the resulting fusion zones from both the front and back sides. Due to the significant microstructural dispersion resulting from the electron beam welding process, scanning electron microscopy was employed for microstructural analysis of the weld and heat-affected zone.

Two process variations were analyzed in the study. In the first variant, only the electron beam welding process was performed, while in the second variant, the samples underwent a heat-treatment process involving stress relieving at 590 °C for 2 h.

The research conducted aimed to address whether the heat-treatment process, specifically stress relieving, carried out after the electron beam welding process of the Ti6Al-4V titanium alloy would significantly affect changes in the alloy’s mechanical properties and microstructure. Additionally, it aimed to determine whether utilizing a lower welding speed than commonly employed would yield a material with the desired properties.

## 2. Test Material Used

The electron beam welding process was carried out for a titanium alloy of grade Ti6Al-4V; the chemical composition is presented in Table 1.

The process was carried out under industrial conditions using a device manufactured by STEIRGERWALD STRAHLTECHNIK GmbH.

Based on preliminary tests, the optimal parameters of the electron beam welding process were determined in order to obtain a weld that would meet the requirements of visual inspection and the parameters of the heat-treatment process, which are presented in Table 2. The heat-treatment process was carried out in a vacuum furnace manufactured by Seco/Warwick.

We conducted an evaluation of the weld shape and size after the electron beam welding.

**Figure 1:**
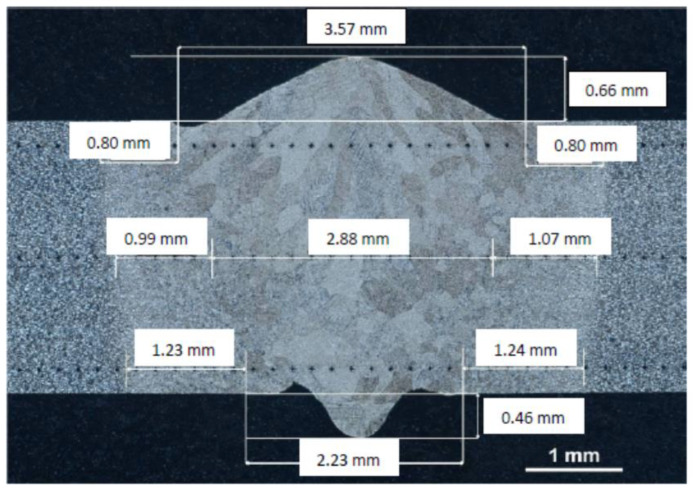
The shape of the weld together with the size of the heat-affected zone. Metallographic analysis using optical microscopy was carried out using an Axiovert 25 optical microscope (Olympus Polska Sp. z o.o., Warsaw, Poland).

Figure 1 shows the shape and size of the weld along with the resulting flooding areas on the face side and the ridge side following the electron beam welding process.

Microstructural analysis after the electron beam welding process revealed that the shape of the butt weld conformed to the requirements of the AWS D1.1 standard [26] in terms of the observed flooding, both from the ridge and the weld face.

The test results also confirmed that the extent of flooding in welds produced using the EBW method did not exceed 10% of the thickness of the welded material.

The welding process yielded a funnel-shaped weld with a maximum width of 3.57 mm on the face side and 2.23 mm on the ridge side.

The height of the weld measured 0.66 mm on the face side and 0.46 mm on the ridge side. These measurements indicated compliance with the standard’s requirements regarding the height of the face and ridge, which must not exceed 50% of the thickness of the welded material.

**Figure 2:**
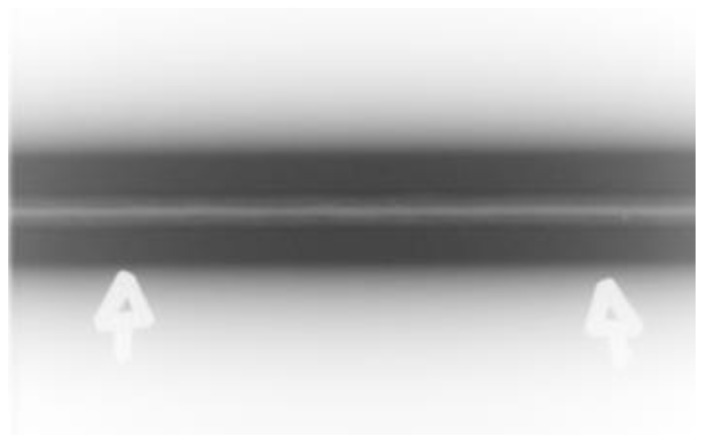
Example of radiograph showing the weld.

Figure 2 displays the heat-affected zones, which exhibited varying widths depending on the distance from the welded surface. The width of the heat-affected zone increased as the distance from the welded surface increased.

## 3. Non-Destructive Testing Analysis

Non-destructive testing of the weld was carried out using the radiographic method (RT) on a Varian NDI-22 device equipped with a Varex Imagine lamp. These tests were carried out in order to evaluate the welded joint obtained in accordance with the parameters established in the tests for the presence of discontinuities and internal defects in it. As a result of the research, several dozen radiographs were obtained. An example radiograph is presented in Figure 2.

On the basis of radiographic analysis of all radiographs obtained in the tests, it was found that there were no internal defects in the welds. These tests confirmed the possibility of obtaining a weld free of discontinuities and internal defects in the process of electron beam welding in accordance with the parameters established in the tests.

## 4. Microhardness Analysis

To comprehensively evaluate the impact of welding parameters and two-stage heat treatment on the mechanical properties of the joined materials, an analysis of the microhardness values was performed at three measurement depths along the cross section of the weld and the heat-affected zone (HAZ), as depicted in Figure 2. The tests were conducted on samples following the electron beam welding process (Variant No. I) and on samples subjected to electron beam welding followed by heat treatment through stress relieving at 590 °C for 2 h (Variant No. II).

**Figure 3:**
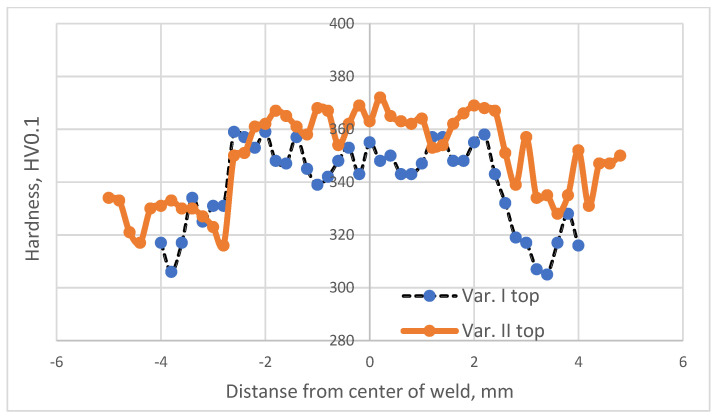
Microhardness distribution for welding Variant Nos. I and II determined for the area at a distance of 0.25 mm from the upper surface of the material.

**Figure 4:**
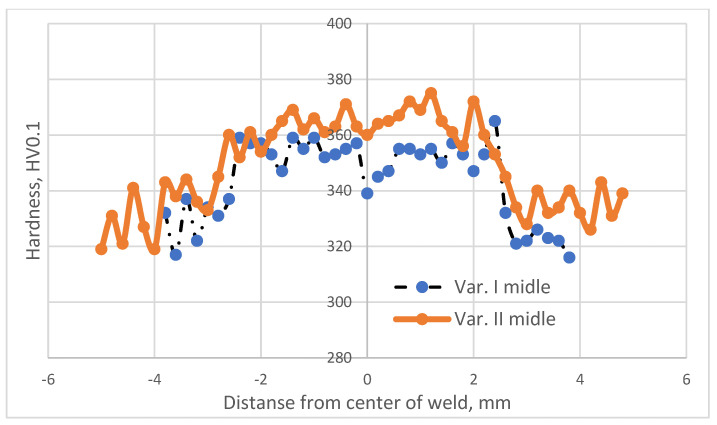
Microhardness distribution for welding Variant Nos. I and II specified in the middle of the weld.

**Figure 5:**
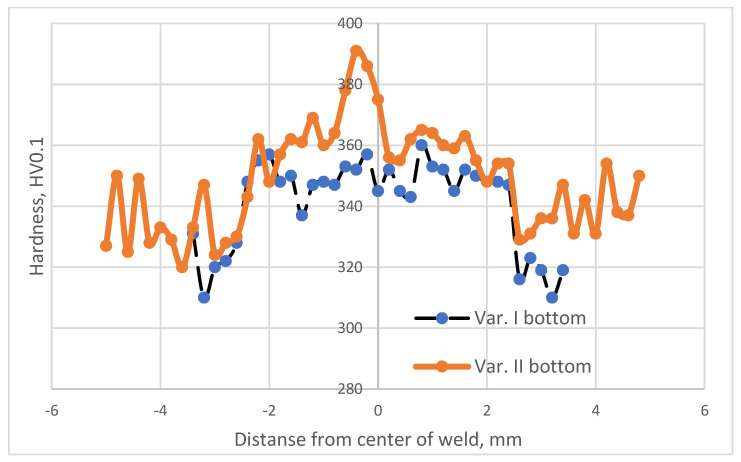
Microhardness distribution for welding Variant Nos. I and II specified for the area at a distance of 0.25 mm from the lower surface of the material.

The study employed a Shimadzu microhardness tester. Figure 3, Figure 4 and Figure 5 illustrate the distribution of microhardness across the cross section of the weld and the heat-affected zone with a focus on the analyzed areas and variants.

After analyzing the distribution of microhardness, it was observed that there was a typical decrease in hardness within the heat-affected zone (HAZ) for all analyzed variants, both in the case of the solely welded material and the material subjected to the EBW process followed by heat treatment.

This reduction in hardness is a common phenomenon in beam welding processes. The additional heat treatment (Variant No. II) did not alter the nature of microhardness distribution within the HAZ; it merely led to a slight increase in its value.

The minimum microhardness values in the heat-affected zone were obtained in Variant I for the area located 0.25 mm from the upper surface of the material (305 HV0.1).

The maximum microhardness values in the heat-affected zone were obtained in Variant II for the area situated 0.25 mm from the upper surface of the material (328 HV0.1).

Analyzing the microhardness distribution within the weld revealed that the heat treatment performed after the electron beam welding process significantly increased the microhardness in this region. It is known from the literature [27] that this is attributed to the precipitation processes occurring from the weld, which was supersaturated during the EBW process.

The minimum microhardness values within the weld were obtained in Variant I for the area located 0.25 mm from the lower surface of the material (337 HV0.1).

The maximum microhardness values within the weld were obtained in Variant II for the area situated 0.25 mm from the lower surface of the material (390 HV0.1).

The heat treatment following the electron beam welding process resulted in an average increase in microhardness within the weld by approximately 4% regardless of the analyzed area.

## 5. Microstructural Analysis of Weld and Heat-Affected Zones

To account for the significant microstructural dispersion resulting from the electron beam welding process, a scanning electron microscopy analysis was conducted using a JSM-5400 scanning microscope (Jeol Akishima, Tokio, Japan) to examine the microstructure of the weld and the heat-affected zones.

**Figure 6:**
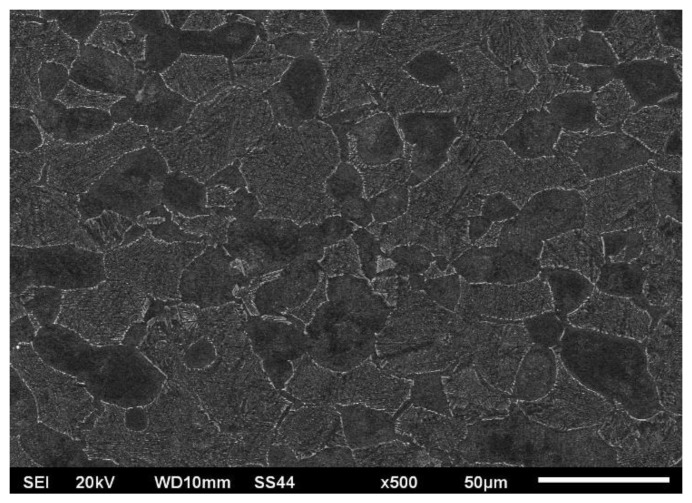
Microstructure of the parent material before the EBW process.

**Figure 7:**
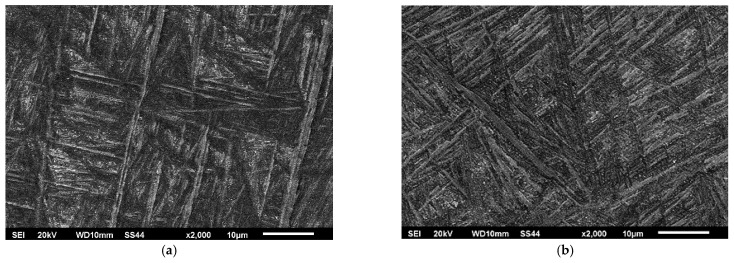
Microstructure center of the weld: (**a**) Variant No. I; (**b**) Variant No. II.

**Figure 8:**
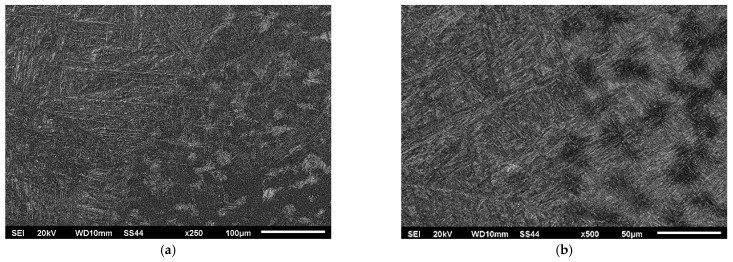
Microstructure of the transition zone weld-hardened in the solid-state zone: (**a**) Variant No I; (**b**) Variant No II.

**Figure 9:**
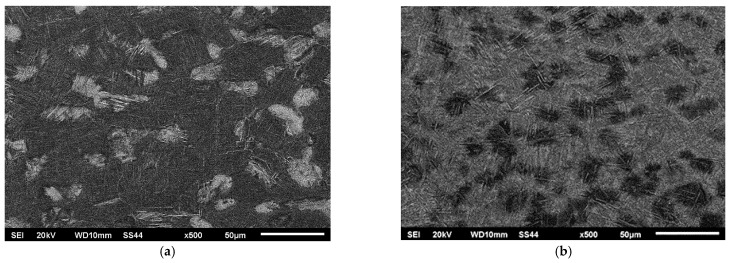
Microstructure of the heat-affected zone (hardened in solid state): (**a**) Variant No I; (**b**) Variant No II.

Figure 6 illustrates the microstructure of the original welded material, while Figure 7, Figure 8 and Figure 9 showcase the macrostructure of the weld, the transition zone, and the heat-affected zone in both variants of the studied processes.

The microstructure of the base material consisted of grains from phase α and a mixture of grains from phases α and β. This structure is considered to be in equilibrium and is commonly observed in the use of Ti6Al-4V titanium alloy for various components in aircraft engines.

During the welding process, the structure underwent supersaturation from the liquid state, resulting in the formation of a coniferous morphology within the weld (Figure 7).

The subsequent heat treatment through stress relief annealing at 590 °C for 2 h did not alter the morphology of the phases within the weld. However, an increase in the presence of fine-dispersed phase precipitates could be observed between the needles in phase α and phase β.

During rapid crystallization, a martensitic transformation occurs, resulting in the presence of a highly defective needle-like structure in the weld. Such a structure is typical for all martensitic structures. In the case of the Ti6Al-4V alloy, the rapid crystallization process in the weld leads to the formation of two types of martensitic phases, namely α’ and α” [28].

Both forms of martensite undergo decomposition through the precipitation of subdispersed β-phase precipitates during post-weld heat treatment. The size of these precipitates depends on the temperature and duration of the process.

In the presented structures within the heat-affected zone, a solid-state martensitic transformation was observed, resulting in the formation of a martensitic structure with clearly different needle dispersion of the martensitic phase.

The purpose of analyzing the microstructure between the weld and the heat-affected zone (HAZ) was to determine if there were specific grain boundaries in the Ti6Al-4V titanium alloy that exhibited a privileged transition to the liquid state during welding.

The microstructures shown in Figure 8 did not indicate any significant influence of the grain boundaries of the Ti6Al-4V titanium alloy on the shaping process of the weld. However, the effect of the initial structure on the process of supersaturation from the solid state was noticeable. The areas where a mixture of phases α and β were present exhibited a higher degree of supersaturation, resulting in the fine-grained coniferous structure seen in Figure 8a and Figure 9a. The grains that were initially in Phase α (visible as lighter areas in Figure 8a and Figure 9a) exhibited a more pear-like coniferous structure.

## 6. Analysis of Weld Chemical Composition

To evaluate the extent of alloying element mixing in the weld after the electron beam welding process, chemical composition studies were conducted using the energy-dispersive X-ray spectroscopy (EDX) method. The research focused on the central part of the weld face and the native material, with measurements taken at four different locations. The findings of the study are presented in Table 3, Table 4 and Table 5.

Based on an analysis of the chemical composition test results for the parent material and the weld after the electron beam welding process, it can be concluded that the welding process conducted under high vacuum conditions resulted in a depletion of aluminum in the titanium alloy at the weld face. Aluminum is an important alloying element that significantly influences the mechanical properties of the final product.

However, the low welding speed employed in the tests (8 mm/s) did not cause the aluminum content to drop below 6.00%, which was the threshold that determined the chemical composition of the Ti6Al-4V alloy and its associated mechanical properties.

This finding was supported by the microhardness values presented in previous studies.

## 7. Analysis of Strength Properties

**Figure 10:**
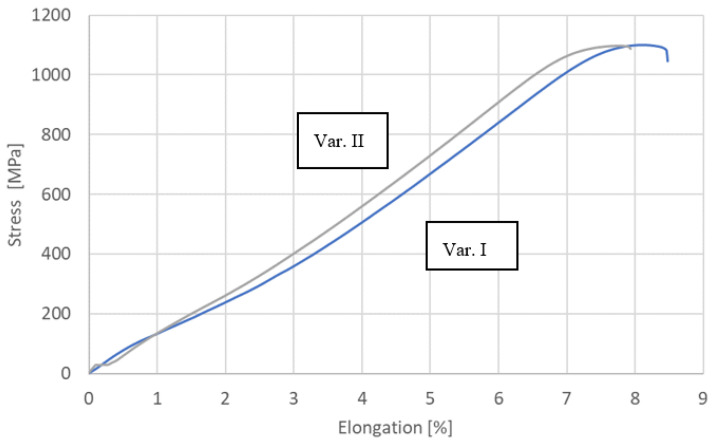
Tensile Curves (Variant Nos. I and II).

Tests of mechanical properties were carried out for all process variants on a ZWICK/Z100 testing machine. These tests were carried out in accordance with the requirements of EN ISO 6892-1 [29]. The results of the research are presented in Figure 10 and Table 6.

**Figure 11:**
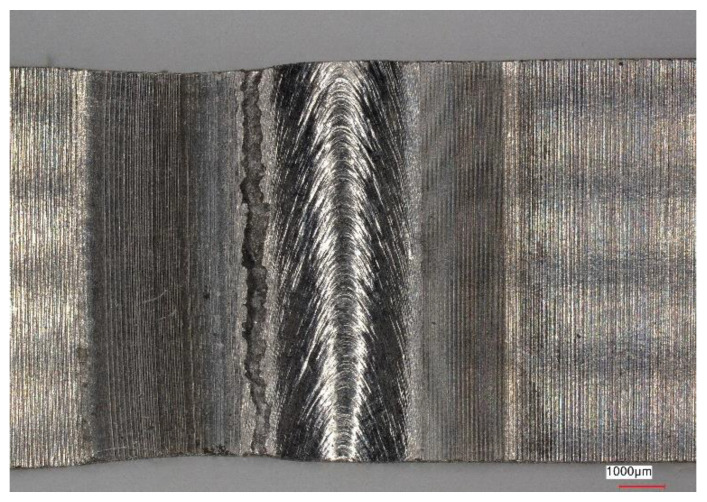
View of the sample (Variant No. I) after the tensile test from the weld face.

**Figure 12:**
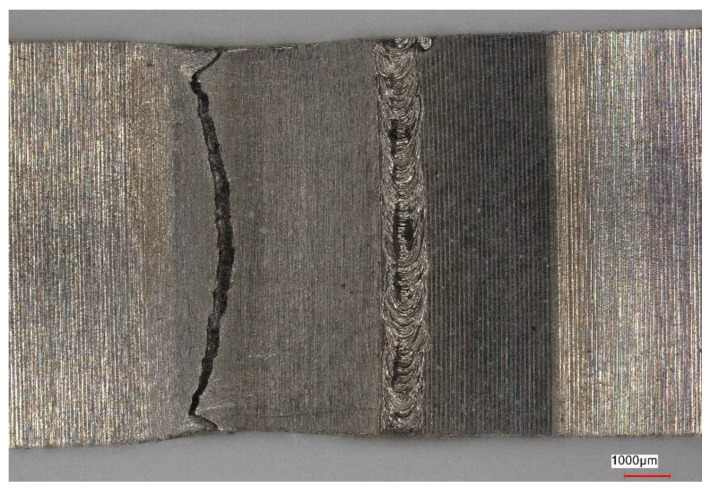
View of the sample (Variant No. I) after the tensile test from the side of the weld ridge.

**Figure 13:**
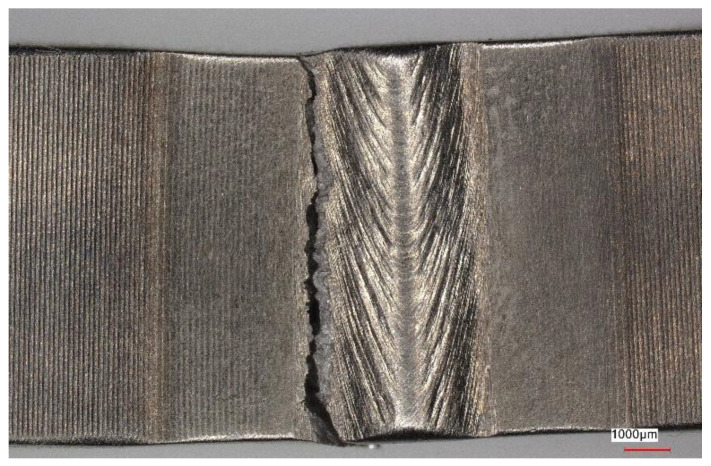
View of the sample (Variant No. II) after the tensile test from the weld face.

**Figure 14:**
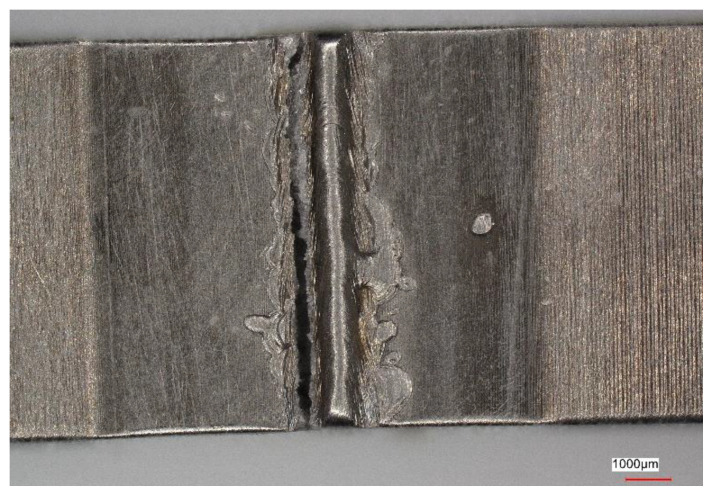
View of the sample (Variant No. II) after the tensile test from the side of the weld ridge.

Figure 11, Figure 12, Figure 13 and Figure 14 show views of the samples after the tensile tests for two variants.

**Figure 15:**
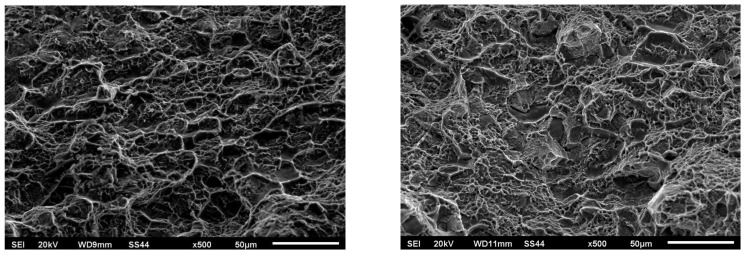
Fractography of the breakthrough after the tensile test for Variant No. I.

**Figure 16:**
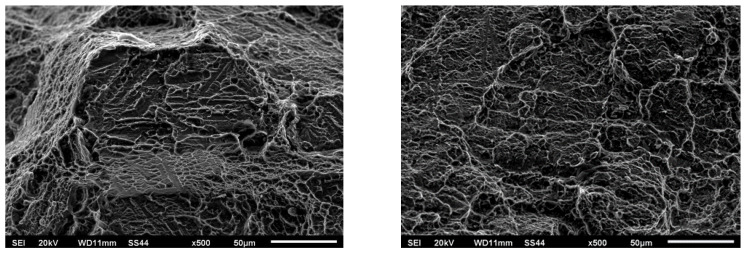
Fractography of the breakthrough after the tensile test for Variant No. II.

Figure 15 and Figure 16 show views of the breakthroughs of the samples after the tensile tests for two variants.

Samples that underwent only the EBW process exhibited a lower yield margin compared to samples that underwent both the EBW process and heat treatment.

Upon analyzing the fracture locations on the face side of the samples, it was observed that for both Variant No. I and Variant No. II, the fractures occurred between the weld and the heat-affected zone (HAZ). However, on the ridge side, in Variant No. I, the fractures occurred along the line between the heat-affected zone and the parent material, while in Variant No. II, the fractures occurred between the heat-affected zone and the weld.

Fractography-based fracture analysis of both variants indicated the absence of a brittle fracture crisis, as cracks did not form in the regions with the lowest microhardness values, which were located away from the weld.

In both cases, a semi-ductile fracture was observed that was characterized by the presence of fine-dispersed separations located within the deformation spaces.

Figure 15 and Figure 16 show numerous cracks, pits, and torn ridges. The fractographic images for both variants exhibit characteristics of ductile fracture. The mechanism of coalescence of micropores during tensile loading was the cause of the observed surface depressions, their size, and depth. This was dependent on the plasticity and strength of the tested material in the fracture region. In Variant No. 1 (Figure 15), the observed cracks were relatively deep and wide and were manifested as large voids. On the other hand, the cracks appearing in Variant No. II (Figure 16) were smaller and shallower. Additionally, in Variant No. II, there was a significant influence of grain boundaries on the fracture mechanism as evidenced by distinct grain boundary markings.

## 8. Conclusions

1. Carrying out the electron beam welding process of Ti6Al-4V-grade titanium alloy at a speed of 8 mm/s allowed for the production of a weld that met the requirements of relevant standards in terms of flooding size, ridge height, and face height. The chosen speed of the EBW process ensured a weld without internal porosity, which is crucial for the suitability of welded components in aircraft engine construction.
2. The subsequent heat-treatment process involving stress relieving at 590 °C for 2 h after the EBW process resulted in an approximate 4% increase in the microhardness compared to the untreated material. This phenomenon was attributed to the precipitation of supersaturated material, which occurred during the welding process, along with dispersions from phase α.
3. No influence of grain boundaries on the resulting fusion line between the weld and the HAZ was observed during the electron beam welding process of the Ti6Al-4V-grade titanium alloy at a speed of 8 mm/s.
4. Implementing the electron beam welding process for the Ti6Al-4V-grade titanium alloy at a speed of 8 mm/s under high vacuum conditions, as used in the study, led to depletion of the material in the face area. This depletion was primarily caused by the evaporation of one of the alloying elements (aluminum) due to its high vaporization tendency. However, reducing the speed of the EBW process did not result in a significant decrease in the aluminum content below the 6% limit, which determined the chemical composition of the alloy and its properties.
5. The mechanical properties of the material after the EBW process alone and after the EBW process followed by heat treatment were comparable, with no differences in tensile strength observed. However, for samples subjected to the EBW process and subsequent heat treatment, a higher yield margin was determined as indicated by the R0.2/R_m_ ratio.
6. The low speed of the electron beam welding process used in the research for the Ti6Al-4V-grade titanium alloy allowed for the production of material with the expected microstructural properties (such as the absence of discontinuities and internal defects) as well as the desired chemical composition.
7. Carrying out the heat-treatment process involving stress relieving of the Ti6Al-4V-grade titanium alloy after the EBW process led to a weld with higher mechanical properties compared to materials that did not undergo the heat-treatment process.

## Figures and Tables

**Table 1:** Chemical composition of titanium alloy grade Ti6Al-4V (% *w*/*w*).

	Al, %	V, %	Fe, %	O, %	C, %	N, %	H, %	Y, %	Ti, %
Min.	5.50	3.50	0	0	0	0	0	0	Remainder
Max.	6.75	4.50	0.30	0.20	0.10	0.05	0.015	0.001	Remainder

**Table 2:** Parameters of the electron beam welding process and heat treatment.

	Welding Current (mA)	Welding Speed (mm/s)	Beam Oscillation (mm)	Beam Defocusing (mA)	Heat-Treatment Temperature (°C)	Heat-Treatment Time (h)	Atmosphere, Pressure (Pa)
Variant I	5.5	8	0	+20	-	-	-
Variant II	5.5	8	0	+20	590	2	Ar, 1000

**Table 3:** Contribution by weight of elements to the chemical composition of the base material.

Measuring Point No.	Element Weight Content in Chemical Composition, %		
	Al	Ti	V
1	7.79	88.78	3.43
2	7.32	88.55	4.12
3	6.5	90.03	3.47
4	8.33	88.09	3.58
Average value	7.48	88.86	3.65

**Table 4:** Contribution by weight of elements to the chemical composition of the face of weld surface (welding speed: 8 mm/s).

Measuring Point No.	Element Weight Content in Chemical Composition, %		
	Al	Ti	V
1	5.51	90.80	3.69
2	6.21	90.18	3.61
3	6.71	89.81	3.47
4	5.86	89.66	4.47
Average value	6.07	90.11	3.81

**Table 5:** Contribution by weight of elements to the chemical composition of the face of weld surface (welding speed: 25 mm/s).

Measuring Point No.	Element Weight Content in Chemical Composition, %		
	Al	Ti	V
1	7.65	88.83	3.52
2	7.44	88.91	3.65
3	7.53	88.30	4.17
4	7.33	89.01	3.66
Average value	7.48	88.76	3.75

**Table 6:** Results of tensile tests.

Variant	R_0.2_, MPa	R_m_, MPa
I	1092	1099
II	1084	1099

## Data Availability

Data sharing is not applicable to this article.
